# Dynamic modulation of phosphoprotein expression in ovarian cancer xenograft models

**DOI:** 10.1186/s12885-016-2212-6

**Published:** 2016-03-10

**Authors:** Antonis Koussounadis, Simon P. Langdon, Inhwa Um, Charlene Kay, Kyle E. Francis, David J. Harrison, V. Anne Smith

**Affiliations:** School of Biology, Sir Harold Mitchell Building, University of St Andrews, St Andrews, Fife KY16 9TH UK; Division of Pathology, Institute of Genetics and Molecular Medicine, University of Edinburgh, Edinburgh, UK; School of Medicine, University of St Andrews, St Andrews, UK

**Keywords:** Ovarian cancer, Carboplatin, Paclitaxel, Xenograft, Prognosis, Phosphoproteins

## Abstract

**Background:**

The dynamic changes that occur in protein expression after treatment of a cancer in vivo are poorly described. In this study we measure the effect of chemotherapy over time on the expression of a panel of proteins in ovarian cancer xenograft models. The objective was to identify phosphoprotein and other protein changes indicative of pathway activation that might link with drug response.

**Methods:**

Two xenograft models, platinum-responsive OV1002 and platinum-unresponsive HOX424, were used. Treatments were carboplatin and carboplatin-paclitaxel. Expression of 49 proteins over 14 days post treatment was measured by quantitative immunofluorescence and analysed by AQUA.

**Results:**

Carboplatin treatment in the platinum-sensitive OV1002 model triggered up-regulation of cell cycle, mTOR and DDR pathways, while at late time points WNT, invasion, EMT and MAPK pathways were modulated. Estrogen receptor-alpha (ESR1) and ERBB pathways were down-regulated early, within 24 h from treatment administration. Combined carboplatin-paclitaxel treatment triggered a more extensive response in the OV1002 model modulating expression of 23 of 49 proteins. Therefore the cell cycle and DDR pathways showed similar or more pronounced changes than with carboplatin alone. In addition to expression of pS6 and pERK increasing, components of the AKT pathway were modulated with pAKT increasing while its regulator PTEN was down-regulated early. WNT signaling, EMT and invasion markers were modulated at later time points. Additional pathways were also observed with the NFκB and JAK/STAT pathways being up-regulated. ESR1 was down-regulated as was HER4, while further protein members of the ERBB pathway were upregulated late. By contrast, in the carboplatin-unresponsive HOX 424 xenograft, carboplatin only modulated expression of MLH1 while carboplatin-paclitaxel treatment modulated ESR1 and pMET.

**Conclusions:**

Thirteen proteins were modulated by carboplatin and a more robust set of changes by carboplatin-paclitaxel. Early changes included DDR and cell cycle regulatory proteins associating with tumor volume changes, as expected. Changes in ESR1 and ERBB signaling were also observed. Late changes included components of MAPK signaling, EMT and invasion markers and coincided in time with reversal in tumor volume reduction. These results suggest potential therapeutic roles for inhibitors of such pathways that may prolong chemotherapeutic effects.

**Electronic supplementary material:**

The online version of this article (doi:10.1186/s12885-016-2212-6) contains supplementary material, which is available to authorized users.

## Background

Ovarian cancer is a leading cause of gynecologic cancer mortality worldwide. Platinum-based drugs, often combined with a taxane, are the main chemotherapy treatment that follows surgical debulking [1]. Although the majority of patients initially respond to this treatment, around 70 % relapse and present with recurrent disease between 6 and 24 months [2]. The overall 5-year survival is around 30 % [3], highlighting the need for improved patient targeting with appropriate treatment. Many treated chemo-resistant patients (30 %) suffer serious side effects needlessly, while their outcomes could be improved by guiding such patients to alternative therapies. Characterisation of the dynamic processes involved in response to chemotherapy in ovarian cancer models may help develop tools for the prediction of chemo-response and outcome and help identify key molecular changes occurring on treatment.

Cancer is the result of alterations in a limited number of pathways [4]. Recent genomic analyses of ovarian cancer have identified several deregulated pathways [5, 6]. For instance cell cycle associated defects are a hallmark of many cancer types including ovarian cancer [7]. Ovarian cancer is characterised by dysregulation of multiple cellular signaling pathways involved in cancer initiation and progression, such as EGFR/ERBB [8], MAPK [9], PI3K/AKT/mTOR [6, 10], WNT [11], JAK/STAT [12], NFκB [13], ERα [14]. Hypoxic response promotes invasiveness by inducing tumor migration and metastasis [15], while SNAIL [16], AKT, EGFR [17] and WNT pathway [18] mediate epithelial-to-mesenchymal (EMT) transition. Several DNA repair pathways, such as ataxia-telangiectasia-mutated (ATM) [19], Fanconi anemia (FA) [20], nucleotide excision repair (NER) pathways [21], are activated in response to platinum-induced DNA damage. A number of these pathways have been associated with chemo-resistance [22], while oncogenic pathway profiles have been associated with clinical outcome [23]. BRCA1/2 mutation status is also informative with respect to platinum response [24].

Activation states of cell signaling pathways can be inferred by measuring expression of representative proteins or phosphoproteins [25]. Quantitative proteomic approaches allow for the estimation of expression of multiple proteins in a large number of samples and have been used in ovarian cancer to identify factors associated with platinum response and to characterise histotype-dependent protein expression [26, 27]. Measurement of multiple proteins over time may reveal dynamic expression patterns of several pathways simultaneously. Clustering tumors according to activation patterns of oncogenic pathways can identify disease relevant deregulation patterns and support molecular classification [28].

In an earlier study, we analysed genome-wide dynamic gene expression changes in ovarian cancer models after chemotherapy treatment [29]. We observed deregulated DNA repair, cell cycle and apoptosis pathways and identified gene sets associated with survival [28]. Here we measured the dynamic effect of carboplatin and paclitaxel chemotherapy on the expression of a combined panel of 49 proteins and phosphoproteins using the same xenograft models. The models had distinct histological origin and diverse response patterns to carboplatin. As in the gene expression study, protein expression was assessed over a period of 14 days after treatment with drugs with concurrent monitoring of tumor volume. The selected proteins are frequently aberrant or belong to oncogenic pathways often deregulated in ovarian cancer [6, 30]. The targeted proteomics approach allowed the simultaneous detection of dynamic oncogenic pathway activation at the protein level in response to treatment, thus providing a complementary aspect to the transcriptomics study. We identified time-related changes in activated oncogenic pathways and we analysed those in respect to tumor volume reduction.

## Methods

### Xenograft dataset

The platinum sensitive OV1002 ovarian cancer xenograft model was derived from a high grade serous adenocarcinoma, while the HOX424 xenograft model was of clear cell/endometrioid origin and had reduced responsiveness to platinum. Both cell lines were established at the Institute of Genetics and Molecular Medicine, University of Edinburgh as described previously [31]. Ovarian tumor fragments were implanted subcutaneously into adult (12 week old) female CD-1 nu/nu mice and were allowed to grow to 4–6 mm in diameter in negative pressure isolators (La Calhene, Cambridge, UK). Mice were then stratified into treatment groups such that mean tumor volumes were equivalent across the groups. Tumor size was monitored twice weekly and relative tumor volumes (RTV) were calculated for each individual tumor by dividing the tumor volume on day t (Vt) by the tumor volume on day 0 (V0). Mice were grouped into three groups: untreated controls, carboplatin treated (50 mg/kg) and carboplatin (50 mg/kg) + paclitaxel treated (10 mg/kg). Treatment was administered as a single intraperitoneal dose on day 0. Groups of both treated and untreated control mice were sacrificed on Day 1, 2, 4, 7 and 14 after treatment. Tumors were placed into formalin, fixed and paraffin embedded. Tissue sections were cut and stained with a range of antibodies as described below against 49 protein targets (Table 1). The xenograft dataset consisted of 99 OV1002 and 67 HOX424 xenograft samples. Phenotypic and raw data are shown in Additional file 1. There were 3–8 biological replicates at each time point and treatment, except at day 2 for HOX424, where there were 2 and 1 replicates for each treatment. Agreement between sample expression levels was good as measured by the correlation coefficients (*r*) among replicates of each condition (mean *r* 0.94, 95 % confidence interval 0.934–0.955, Additional file 2, column E). The xenograft studies were undertaken under a UK Home Office Project Licence in accordance with the Animals (Scientific Procedures) Act 1986 and studies were approved by the University of Edinburgh Animal Ethics Committee.

**Table 1:** Protein targets

Entrez Gene ID	Gene symbol	Protein (phospho-protein)	Gene name	Function
1017	*CDK2*	CDK2, pCDK2	cyclin-dependent kinase 2	CELL CYCLE
891	*CCNB1*	CyclinB1	cyclin B1	CELL CYCLE
595	*CCND1*	CyclinD1	cyclin D1	CELL CYCLE
4609	*MYC*	Myc	v-myc myelocytomatosis viral oncogene homolog (avian)	CELL CYCLE
1026	*CDKN1A*	P21	cyclin-dependent kinase inhibitor 1A (p21, Cip1)	CELL CYCLE
1027	*CDKN1B*	P27	cyclin-dependent kinase inhibitor 1B (p27, Kip1)	CELL CYCLE
5925	*RB1*	pRb	retinoblastoma 1	CELL CYCLE
672	*BRCA1*	BRCA1, pBRCA1	breast cancer 1, early onset	DNA DAMAGE REPAIR
2067	*ERCC1*	ERCC1	excision repair cross-complementing rodent repair deficiency, complementation group 1	DNA DAMAGE REPAIR
4292	*MLH1*	MLH1	mutL homolog 1, colon cancer, nonpolyposis type 2 (E. coli)	DNA DAMAGE REPAIR
4436	*MSH2*	MSH2	mutS homolog 2, colon cancer, nonpolyposis type 1 (E. coli)	DNA DAMAGE REPAIR
2956	*MSH6*	MSH6	mutS homolog 6 (E. coli)	DNA DAMAGE REPAIR
1111	*CHEK1*	pChk1	CHK1 checkpoint homolog (S. pombe)	DNA DAMAGE REPAIR
3014	*H2AFX*	pH2AX	H2A histone family, member X	DNA DAMAGE REPAIR
3021	*H3F3B*	pHH3	H3 histone, family 3B (H3.3B)	DNA DAMAGE REPAIR
5395	*PMS2*	PMS2	PMS2 postmeiotic segregation increased 2 (S. cerevisiae)	DNA DAMAGE REPAIR
7157	*TP53*	pP53	tumor protein p53	DNA DAMAGE REPAIR
8202	*NCOA3*	AIB1	nuclear receptor coactivator 3	GROWTH SIGNALING:MAPK signaling
207	*AKT1*	AKT, pAKT	v-akt murine thymoma viral oncogene homolog 1	GROWTH SIGNALING:PI3K/AKT signaling
1499	*CTNNB1*	BCatenin, pBCatenin	catenin (cadherin-associated protein), beta 1, 88 kDa	GROWTH SIGNALING:Wnt signaling
1956	*EGFR*	EGFR	epidermal growth factor receptor (erythroblastic leukemia viral (v-erb-b) oncogene homolog, avian)	GROWTH SIGNALING:ERBB signaling
2099	*ESR1*	ER,pER	estrogen receptor 1	GROWTH SIGNALING:ESR signaling
5594	*MAPK1*	ERK, pERK	mitogen-activated protein kinase 1	GROWTH SIGNALING:MAPK signaling
2064	*ERBB2*	HER2	v-erb-b2 erythroblastic leukemia viral oncogene homolog 2, neuro/glioblastoma derived oncogene homolog (avian)	GROWTH SIGNALING:ERBB signaling
2065	*ERBB3*	HER3	v-erb-b2 erythroblastic leukemia viral oncogene homolog 3 (avian)	GROWTH SIGNALING:ERBB signaling
2066	*ERBB4*	HER4	v-erb-a erythroblastic leukemia viral oncogene homolog 4 (avian)	GROWTH SIGNALING:ERBB signaling
3486	*IGFBP3*	IGFBP3	insulin-like growth factor binding protein 3	GROWTH SIGNALING: IGF signaling
3488	*IGFBP5*	IGFBP5	insulin-like growth factor binding protein 5	GROWTH SIGNALING: IGF signaling
6194	*RPS6*	S6, pS6	ribosomal protein S6	GROWTH SIGNALING:mTOR signaling, hypoxia
5241	*PGR*	PgR	progesterone receptor	GROWTH SIGNALING:PI3K/AKT signaling
5604	*MAP2K1*	pMEK	mitogen-activated protein kinase kinase 1	GROWTH SIGNALING:MAPK signaling
4790	*NFKB1*	pNFkB	nuclear factor of kappa light polypeptide gene enhancer in B-cells 1	GROWTH SIGNALING:NFkB signaling
6774	*STAT3*	pSTAT3	signal transducer and activator of transcription 3	GROWTH SIGNALING:Jak/STAT signaling
5728	*PTEN*	PTEN	phosphatase and tensin homolog	GROWTH SIGNALING:PI3K/AKT signaling
1366	*CLDN7*	Claudin7	claudin 7	ETM/INVASION
999	*CDH1*	Ecadherin	cadherin 1, type 1, E-cadherin (epithelial)	ETM/INVASION
3091	*HIF1A*	HIF1alpha	hypoxia inducible factor 1, alpha subunit	ETM/INVASION
4233	*MET*	MET, pMET	met proto-oncogene	ETM/INVASION
5054	*SERPINE1*	PAI	serpin peptidase inhibitor, clade E (nexin, plasminogen activator inhibitor type 1), member 1	ETM/INVASION
6615	*SNAI1*	Snail	snail homolog 1 (Drosophila)	ETM/INVASION
7431	*VIM*	Vimentin	vimentin	ETM/INVASION

Details and function of 49 protein form targets (41 proteins) and their pathways tested in the xenograft protein expression experiment. There were 16 phosphorylated proteins, 8 of which were tested for both phosphorylated and unphosphorylated forms

### Protein expression

Protein expression was measured by immunofluorescence using methods previously described [25]. Tissue microarrays were prepared from xenograft material that had been formalin-fixed at the time of collection and had been processed into paraffin blocks. Microarray slides were deparaffinized and antigen-retrieved by pressure-cooking. Endogenous peroxidases were blocked with 2.5 % hydrogen peroxide for 15 min and non-specific binding blocked with serum-free protein block for 15 min. Slides were then incubated with primary antibodies (Table 2) with AE1/AE3 mouse monoclonal cytokeratin antibody at room temperature for 1 h. After washing, sections were incubated for 1 h at room temperature with secondary antibodies, which included an Alexa 555-conjugated goat anti-mouse antibody diluted 1:100, and prediluted goat anti-rabbit antibody conjugated to a horseradish peroxidase decorated dextran-polymer backbone (EnVision, Dako). Slides were then incubated for 10 min with Cy5-tyramide, which is activated by horseradish peroxidase, to visualise target protein expression. 4', 6-Diamidino-2-phenylindole (DAPI; Molecular Probes, Eugene, Ore) was used to stain the nuclear compartment. For analysis, pan-cytokeratin antibody was used to identify tumor cells and normal epithelial cells, DAPI counterstain to identify nuclei, and Cy-5-tyramide detection for target protein in compartmentalised (tissue and subcellular) analysis of tissue sections. Monochromatic images of each 0.6 mm TMA core were captured at 20× objective using an Olympus AX-51 epifluorescence microscope, and high-resolution digital images analysed by the AQUAnalysis software. AQUA scores, which represent the sum of the Cy5-tyramide score for the target protein divided by the area of the cellular compartment (cytoplasm or nucleus), were then generated for each sample. A detailed description of the AQUA analysis has been reported elsewhere [32].

**Table 2:** Sources of antibodies and the dilutions used in this study

Protein name	Gene name	Source	Catalog No.	Dilution
AIB1	*NCOA3*	BD Biosciences	61105	1 in 50
AKT1	*AKT*	Cell Signaling	Ab4685	1 in 200
pAKT	*AKT*	Cell Signaling	9271	1 in 50
BRCA1	*BRCA1*	Eurogentec	75460	1 in 70
pBRCA1	*BRCA1*	Cell Signaling	9009	1 in 25
CyclinB1	*CCNB1*	Epitomics	1495-1	1 in 50
CyclinD1	*CCND1*	Dako	M3635	1 in 100
E-Cadherin	*CDH1*	BD sciences	610181	1 in 1500
CDK2	*CDK2*	Cell Signaling	2546	1 in 100
pCDK2	*CDK2*	Cell Signaling	2561	1 in 1500
CDKN1A	*P21*	Cell Signaling	2946	1 in 100
CDKN1B	*P27*	Cell Signaling	2552	1 in 100
CLDN7	*Claudin7*	Abcam	AB27487	1 in 200
B-Catenin	*CTNNB1*	BD sciences	610153	1 in 500
pB-Catenin	*CTNNB1*	Cell Signaling	9561	1 in 25
EGFR	*EGFR*	Invitrogen	28-0005	1 in 50
ERBB2	*HER2*	Dako	A0485	1 in 400
ERBB3	*HER3*	Dako	M7297	1 in 50
ERCC1	*ERCC1*	Labvision	MS-671-P0	1 in 600
ER1	*ESR1*	Neomarkers	RM-9101-S1	1 in 100
pER1	*ESR1*	Cell Signaling	2511	1 in 50
pH2AX	*H2AFX*	Cell Signaling	2577	1 in 50
HER2	*HER2*	Dako	M7297	1 in 400
HER3	*HER3*	Cell Signaling	4754	1 in 50
HER4	*HER4*	Cell Signaling	4792	1 in 300
HIF1A	*HIF1a*	Cell Signaling	3176	1 in 25
IGFBP3	*IGFBP3*	Abcam	Ab4248	1 in 100
IGFBP5	*IGFBP5*	Abcam	Ab4255	1 in 50
MAPK1	*ERK*	Cell Signaling	9107	1 in 250
pMEK	*MAP2K1*	Cell Signaling	9154	1 in 400
MET	*MET*	Eurogentec	75551	1 in 400
pMET	*MET*	Eurogentec	65559	1 in 25
MLH1	*MLH1*	Leica	NCL-L-MLH1	1 in 100
MSH2	*MSH2*	Invitrogen	33-7900	1 in 50
MSH6	*MSH6*	Leica	NCL-L-MSH6	1 in 250
MYC	*MYC*	Eurogentec	75355	1 in 70
pNFKB	*NFKB1*	Cell Signaling	3037	1 in 25
PAI	*SERPINE1*	BD Sciences	P612024	1 in 200
PgR	*PGR*	Dako	3569	1 in 50
PMS2	*PMS2*	Leica	NCL-L-PMS2	1 in 250
pP53	*TP53*	Cell Signaling	9286	1 in 100
PTEN	*PTEN*	Cell Signaling	9559	1 in 200
pRB	*RB1*	Cell Signaling	9308	1 in 50
S6	*RPS6*	Cell Signaling	2217	1 in 100
pS6	*RPS6*	Cell Signaling	2211	1 in 200
SERPINE1	*PAI*	BD Sciences	P612024	1 in 200
SNAIL	*SNAI1*	Abcam	Ab17732	1 in 800
pSTAT3	*STAT3*	Cell Signaling	9145	1 in 25
Vimentin	*VIM*	Sigma	V6630	1 in 400

### Protein expression analysis and statistics

AQUA scores were log-transformed averaged from replicate cores. Means of differentially expressed proteins log fold change values for each time point were hierarchically clustered using Cluster 3.0 in order to identify significant proteins with similar temporal expression profiles. Heatmaps were visualized using TreeView [33]. Bioconductor package *limma* [34] was used for differential expression calculations. Significant genes had FDR adjusted *p*-values below 0.05. Treated samples were contrasted to pooled untreated control from all time points in each xenograft. Differentially expressed proteins were classified as expressed early (Days 1–4) or late (Days 7, 14), and transient (expressed significantly in 1 time point) or continuous (expressed significantly at least 2 continuous time points).

## Results

### Carboplatin dynamically activates phosphoproteins in ovarian cancer xenografts

**Fig. 1:**
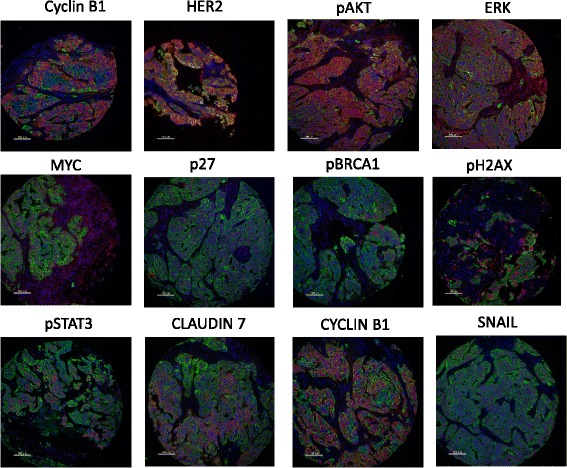
Stained tissue images for AQUA quantitative image analysis. Representative immunofluorescence images of examples of the phosphoproteins or total proteins used within the study. *Blue* = DAPI nuclear counterstain, *Green* = cytokeratin tumor mask, *Red* = target protein. Formaldehyde fixed paraffin-embedded xenograft tissue was stained and analysed as described in Methods

Protein candidates were selected from pathways identified in a recent analysis of somatic mutations and copy number changes in ovarian cancer [5] and known pathways of interest in this disease [25]. A total of 49 protein forms were investigated which are known to be involved in DDR (BRCA1/pBRCA1, ERCC1, MLH1, MSH2, MSH6, pCHK1, pH2AX, PHH3, PMS2, pP53), cell cycle regulation (CDK2/pCDK2, Cyclin B, Cyclin D1, MYC, p21, p27, pRb), EMT/invasion (Claudin 7, E-cadherin, HIF1α, MET/pMET, PAI1, SNAIL, Vimentin) and growth signaling including WNT (CTNNB1/pCTNNB1), PI3K/AKT (AKT/pAKT, PTEN), mTOR (S6/pS6), NFκB (NFκB1), MAPK (ERK1/pERK1, pMEK), EGFR/ERBB (EGFR, HER2, HER3, HER4), IGF (IGFBP3, IGFBP5), JAK/STAT (pSTAT3) and ERα (ESR1/pESR1, PGR, AIB1) pathways. Further details of protein targets are shown in Table 1. Examples of stained images are illustrated in Fig. 1. There were 41 unique proteins in the set, 8 of which were in both native and phosphorylated forms and 8 only in phosphorylated form. Activated pathways would feature increased expression of phosphorylated proteins, usually coupled with a concurrent drop in expression of the native form.

**Fig. 2:**
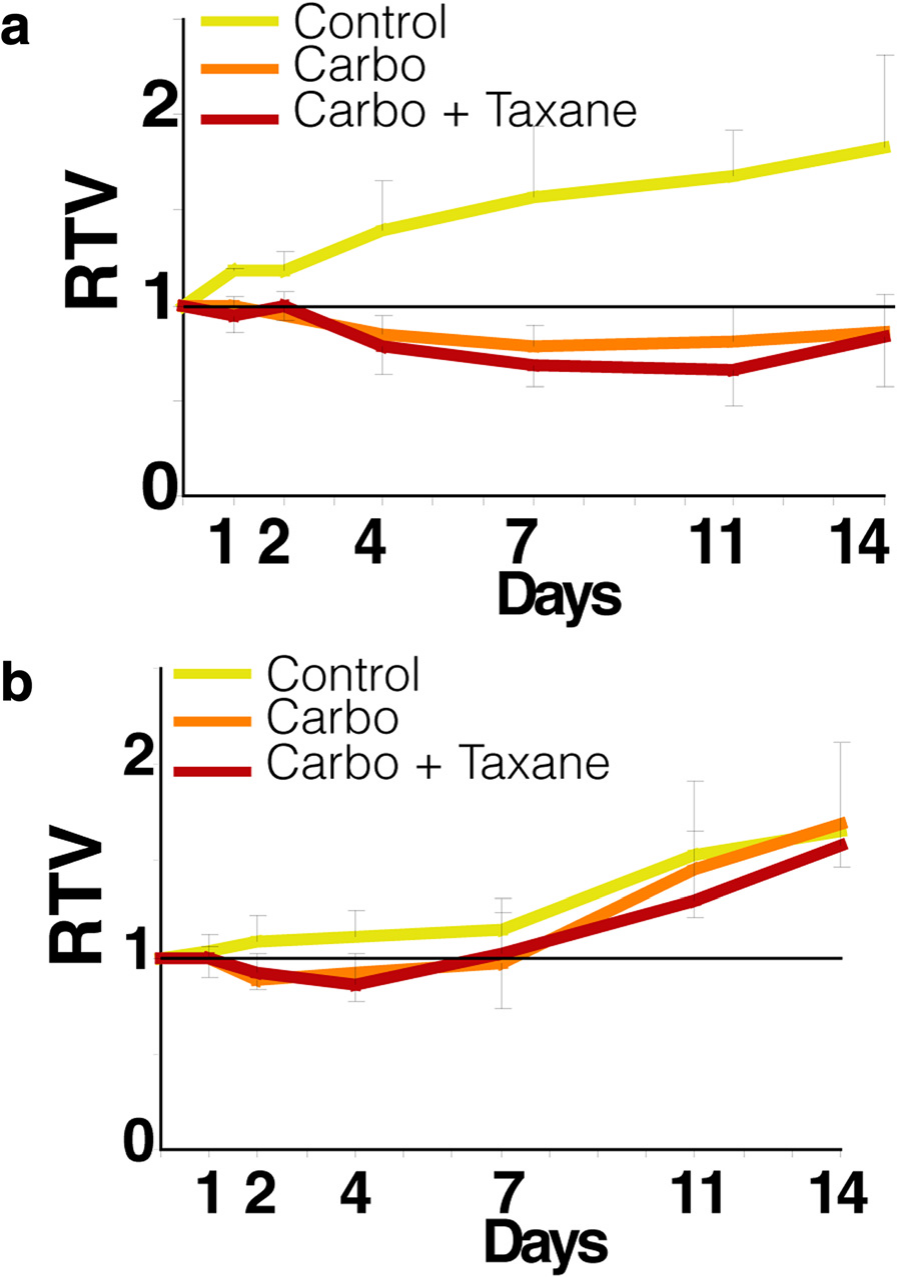
Relative Tumor Volume graphs. Post-treatment tumor volume growth relative to day of treatment (Day 0) for the OV1002 (**a**) and HOX424 (**b**) xenografts. (Carbo: Carboplatin; Taxane: Paclitaxel). Modified from [29]

**Fig. 3:**
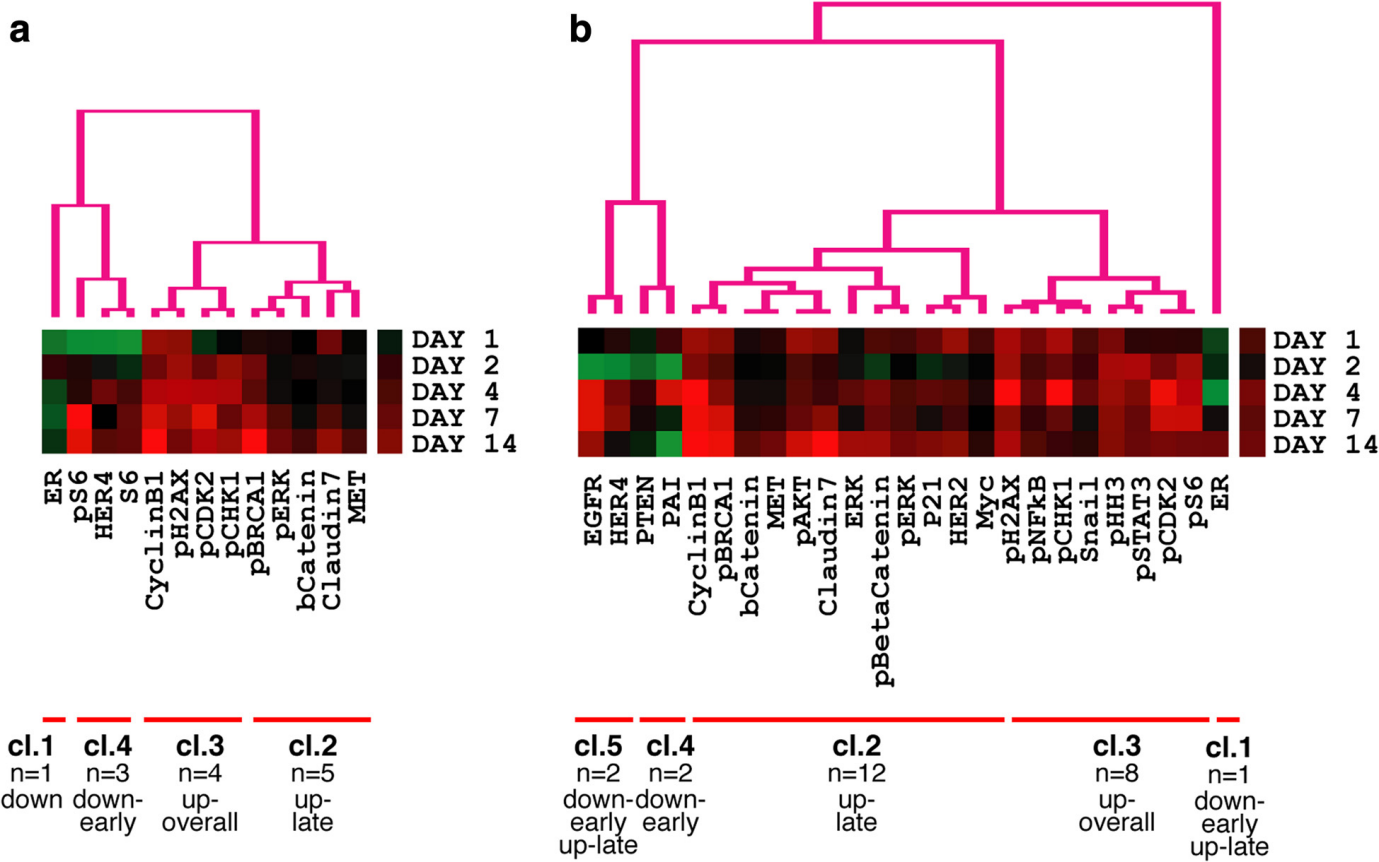
Hierarchical clustering of post-treatment dynamic phosphoprotein expression. Heatmaps illustrating clusters of proteins with differentially increased (*red*) or decreased *(green*) expression in response to carboplatin (**a**) or carboplatin-paclitaxel (**b**) treatment in OV1002 xenografts

**Fig. 4:**
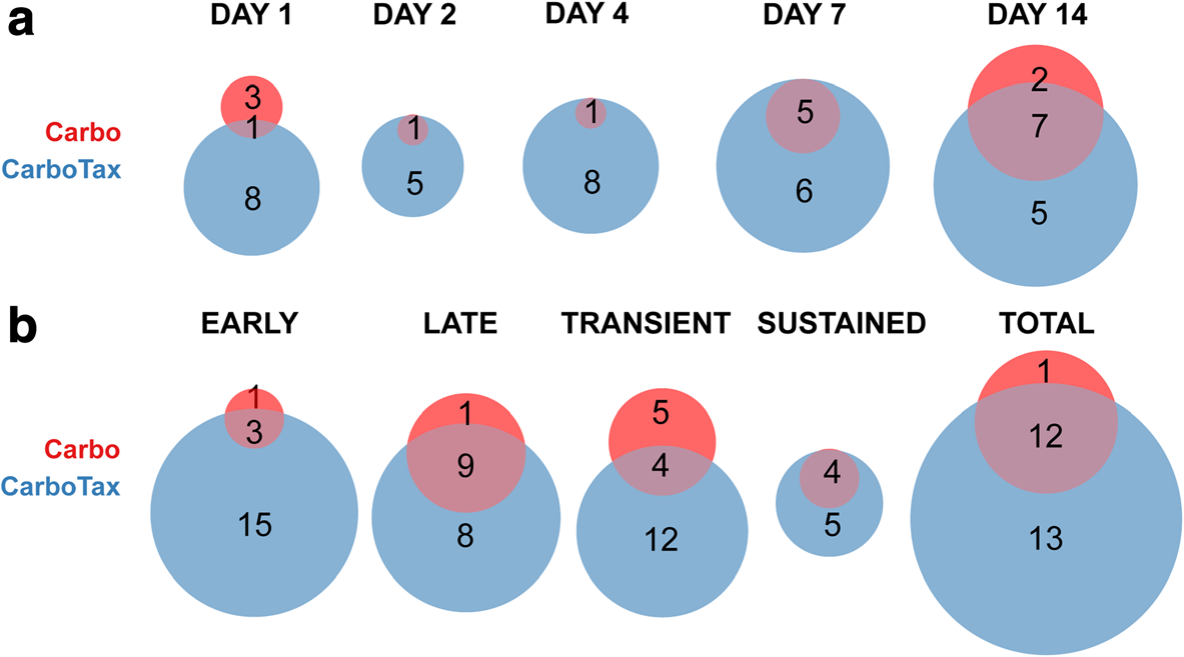
Differential expression analysis of treated OV1002 xenografts. Venn diagrams of differentially expressed protein form counts in each time point (**a**) and according to their temporal characteristics (**b**) in carboplatin and carboplatin-paclitaxel treated OV1002 xenografts. Protein expression was analysed in 5 time points post treatment (Days 1, 2, 4, 7 and 14). Early (late) genes were differentially expressed in Days 1, 2 and 4 (Days 7 and 14). Transient (sustained) were differentially expressed in a single time point (more than one time points)

**Fig. 5:**
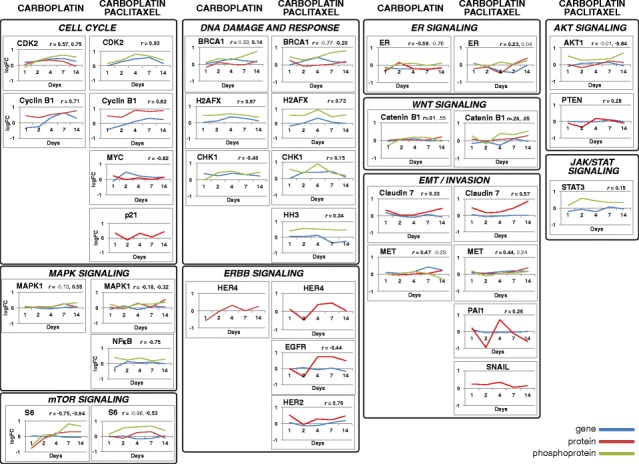
Post-treatment dynamic phosphoprotein/native protein differential expression graphs. Mean log fold differential expression of phosphoproteins (*orange*)/native proteins (*red*) in treated OV1002 xenografts as compared to untreated controls. Proteins are significantly expressed in at least one time point. Corresponding gene expression from [29] (GEO accession number GSE49577) is shown in blue where available, and its correlation to protein expression is indicated

We have previously shown significant relative tumor volume reduction within the period up to 14 days after a single treatment in the platinum responsive OV1002 and a minimal response in platinum resistant HOX424 ovarian cancer xenograft models [29] (Fig. 2). This same sample set was analysed for the above-named proteins. Carboplatin was administered at 50 mg/kg and tumors collected on days 1, 2, 4, 7 and 14 after treatment. Over this time period, analysis of the protein set identified 13 differentially expressed protein forms (ESR1, βCatenin, Claudin 7, MET, Cyclin B1, HER4, S6, pBRCA1, pERK, pCDK2, pCHK1, pH2AX, pS6) in carboplatin treated OV1002 xenografts, of which 6 were phosphoproteins (Additional file 3). Proteins were grouped by hierarchical clustering into 3 clusters (which we have identified as downregulated early, upregulated late, and upregulated overall, corresponding to their dynamic changes) and a singleton (ESR1 - which was downregulated) (Fig. 3a). Differential expression was mostly late and transient (Fig. 4). Native S6 was reduced early and substituted by its late expressed phosphorylated form (pS6) over time. pCDK2, pERK, pBRCA1 were upregulated at late time points, while expression of their unphosphorylated counterparts was not significantly changed (Fig. 5). Phosphorylated pH2AX was differentially expressed at all five time points, while pCHK1 only at day 14.

In contrast to the increased phosphoprotein expression in treated xenografts, a range of unphosphorylated protein expression responses was observed. Expression of HER4, S6 and ER was inhibited one day post treatment, while MET, βCatenin, Claudin7 and CyclinB1 were upregulated late (Fig. 5). Four of these proteins were found differentially expressed also in the gene level: CDK2, Cyclin B1, CHK1 and MET [29].

The HOX424 response to treatment was very limited. Only one protein (MLH1) was differentially expressed in carboplatin-treated xenografts (Additional file 3). This minimal response is consistent with the very limited tumor growth response to carboplatin (Additional file 3).

### Carboplatin-paclitaxel dynamically trigger a broader and stronger phosphoprotein expression in ovarian cancer xenografts

The combined carboplatin-paclitaxel treatment triggered a more extensive response than carboplatin alone in the OV1002 model. In contrast to only 13 proteins showing differential expression with carboplatin, a total of 23 proteins (25 protein forms) (EGFR, HER4, PTEN, PAI, Cyclin B1, pBRCA1, CTNNB1, MET, pAKT, Claudin 7, ERK, pCTNNB1, pERK, p21, HER2, MYC, pH2AX, pNFκB, pCHK1, SNAIL, pHH3, pSTAT3, pCDK2, pS6, ESR1) were differentially expressed at both early and late time points, of which 11 were phosphoproteins (Figs. 4 and 5, Additional file 3). These were clustered by hierarchical clustering into 4 groups (identified as upregulated late, upregulated overall, downregulated early, down- early/upregulated late) and a singleton (ESR1 - downregulated) (Fig. 3b). Differentially expressed proteins included all but one (S6) that were found significant after carboplatin only treatment. Longitudinal expression profiles between treatments were similar, especially among phosphoproteins. Some distinct expression patterns were observed after carboplatin-paclitaxel treatment. Expression of pBRCA1 was increased immediately after treatment (day 1), while pCHK1 had a higher log fold change across all time points compared to carboplatin only treatment. Five additional phosphoproteins were differentially expressed only after combined treatment. Expression of pAKT and pNFκB were triggered shortly after treatment (day 1), followed by pSTAT3 and pHH3 which were differentially expressed from day 2. Phospho-βCatenin was expressed late, from 7 days after treatment (Fig. 5). Expression of four phosphoproteins was changed at least 2-fold: pH2AX, pCHK1, pCDK2 and pBRCA1 (Fig. 5).

In contrast to carboplatin only treatment, early HER4 and ERα down-regulation were followed by up-regulation at late time points after combination treatment. Additionally, up-regulation of Cyclin B1 and Claudin 7 started immediately after treatment. Another eight proteins were differentially expressed after carboplatin-paclitaxel treatment. Expression of PAI and PTEN was inhibited at day 2. Expression of HER2 and MYC was upregulated at day 1 only, while EGFR and ERK were upregulated late. Snail and p21 were upregulated at early and late time points (Fig. 4). CDK2, Cyclin B1, BRCA1, CHK1, ESR1, HH3 and STAT3 were differentially expressed in the corresponding treatment to control comparison in the gene expression study [29]. All were upregulated in both gene and protein level, except the last two which were downregulated in the gene level and positively regulated in the protein level. Protein expression of HH3 and STAT3 was assessed only in the phosphorylated form and this may explain the discrepancy in expression between gene and protein.

The HOX424 response to combination treatment, as with carboplatin alone treatment, was very limited. Only two proteins (ESR1 and pMET) were differentially expressed in carboplatin-paclitaxel treated xenografts (Additional file 3). Again this is consistent with the very minimal growth response shown to these drugs (Fig. 2).

## Discussion

In this study, we characterised a dynamic protein response to chemotherapy in two ovarian cancer models over a period of 14 days using a set of protein markers of pathways that are frequently deregulated in cancer and may be associated with survival. Complementing an earlier gene expression study, we found multiple proteins differentially expressed after treatment in either or both their native and phosphorylated forms allowing assessment of relative expression of each form over time. An increase of the activated (phosphorylated) forms was observed indicating the activation of several pathways in response to treatment. Certain proteins showed late change in expression, detected here due to the relatively prolonged period of expression monitoring (14 days). There is potential to collect serial samples during treatment of ovarian cancer, e.g., during neoadjuvant therapy which is being increasingly considered prior to surgery [35] or during adjuvant therapy by studying peritoneal ascitic cells removed from patients before and after therapy. Measurement of the dynamic markers identified within this study could provide further information to aid in the assessment of whether the tumor is sensitive to treatment or not.

We observed distinct responses to treatment in each cell line xenograft model. The serous derived OV1002 xenograft was highly responsive to carboplatin-based treatment as judged by a decrease in RTV and marked differential protein expression analysis. Differential protein expression in the slow growing and mainly unresponsive clear cell/endometrioid derived HOX424 was very limited after treatment, while the drop in RTV was smaller in duration and magnitude. This observation coincided with the post-treatment differential expression pattern of these models at the gene level [29]. Correlation of gene and protein expression was higher when both were differentially expressed [36]. These results suggest that responses to treatment among ovarian cancer models of distinct histological origin vary which is consistent with clinical experience. Testing more models of diverse origin would be valuable in helping to assess the range of responses to treatment in ovarian cancer.

Carboplatin treatment in the platinum-sensitive OV1002 model triggered up-regulation of cell cycle (Cyclin B1, pCDK2, pCHK1), mTOR (S6, pS6) and DRR (pH2AX, pBRCA1, pCHK1) pathways, while at late time points WNT (βCatenin), invasion (Claudin 7), EMT (c-MET) and MAPK (pERK) pathways were modulated. Estrogen receptor-alpha (ESR1) and ERBB (HER4) pathways were down-regulated early, within 24 h, as was S6. The early increase in pH2AX at 24 h was maintained through to Day 7 after which it fell. Cell cycle changes (pCDK2, Cyclin B1) increased progressively over the first 7 days and at the latest time point indicators of WNT, EMT, Invasion and MAPK pathways were increased. The changes in DNA damage response and repair proteins and in cell cycle associated proteins are in line with the DNA lesions produced by carboplatin and consistent with its mode of action [37]. In platinum-sensitive ovarian cancer cells in vitro, we have previously shown that markers of DDR such as pBRCA1 and pɣH2AX are known to be induced over a 3–4 day period comparable to that seen in vivo [38]. The changes observed in the current study are also consistent with differential expression patterns observed in the gene expression study, where several cell cycle and DDR proteins used here were significant (Fig. 5). These variations were anticipated and match the changes in tumor volume after treatment.

Phosphorylation of H2AX on Ser139 is consistent with formation of DNA strand breaks [39], while BRCA1 is phosphorylated by ATM in response to double-stranded DNA breaks [40]. CHK1 Ser 317 phosphorylation is then consistent with checkpoint activation [41]. βCatenin has been proposed as a target in this disease and may be associated with chemoresistance [18]. Claudin 7 is frequently overexpressed in ovarian cancer and has been linked to increased migration [42]. High expression of c-MET in ovarian cancer has been associated with poor survival and the use of c-MET inhibitors could enhance the effects of platinum-taxane therapy [43, 44]. The early changes in ESR1 and HER4 were unexpected as were the later changes in invasion and EMT proteins. High expression of ESR1 has been associated with outcome for ovarian cancer after platinum-based therapy [45] and also decreased on carboplatin treatment. Expression of HER4 (ERBB4) has been associated with chemoresistance [46] in ovarian cancer and this decreased on carboplatin treatment. Several of the later changes may reflect rapid regrowth, for example the increases in pERK and pAKT. These proteins are key in activation of their corresponding ERK and AKT pathways which are complex, interacting cascades playing key roles in normal and malignant cell growth [47].

**Fig. 6:**
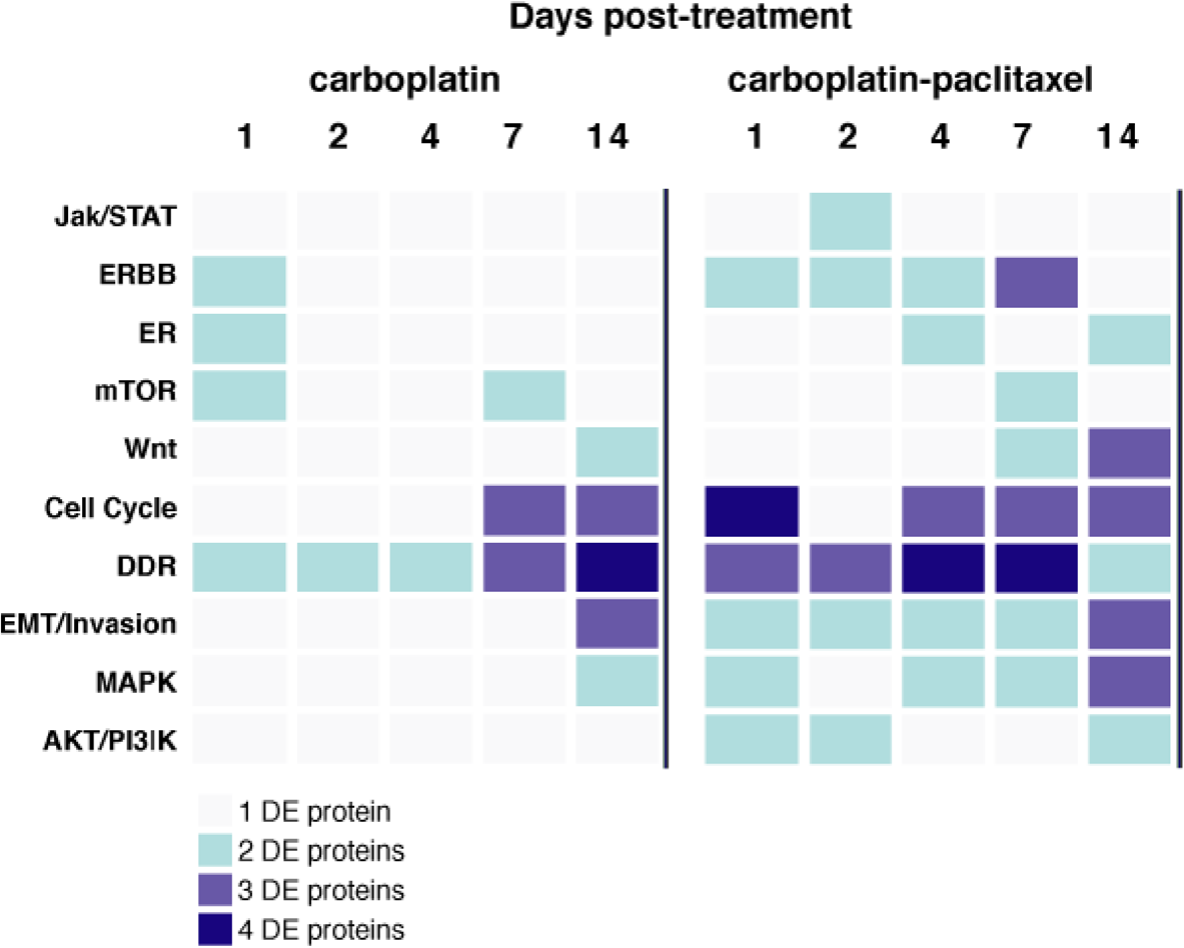
Heatmap of post-treatment dynamic pathway activation in OV1002 xenografts. The number of differentially expressed (DE) protein members of a pathway (Jak/STAT signaling, ERBB signaling, ERα signaling, mTOR signaling, WNT signaling, cell cycle, DDR, EMT/Invasion, MAPK signaling and AKT signaling) in each time point is colour coded in shades of blue, for each treatment: carboplatin and carboplatin-paclitaxel

Further responses were observed when carboplatin-paclitaxel combination therapy was compared with carboplatin treatment alone. The dynamic response to the carboplatin-paclitaxel combination involved similar or greater responses in the same pathways but with more proteins demonstrating a response while additional pathway molecules were also modulated (Fig. 6). Therefore the cell cycle (Cyclin B1, pCDK2, pCHK1 and also pHH3, MYC and p21) and DDR (pH2AX, pBRCA1, pCHK1) pathways showed similar or more pronounced changes than with carboplatin alone and with more of the tested proteins demonstrating changed expression. Consistent to our findings, p21 (CDKN1A) is reported to increase after paclitaxel treatment in ovarian cancer cells contributing to cell cycle regulation [48]. In addition to the mTOR molecule pS6 increasing, components of the AKT pathway (pAKT, PTEN) were modulated with pAKT increasing, while its tumor suppressor regulator PTEN was down-regulated early on. The MAPK pathway was again modulated with pERK expression increasing. WNT signaling (both βCatenin and phospho-βCatenin), EMT markers (MET, SNAI1), invasion (Claudin 7 and PAI) were again modulated generally at later time points. Increased SNAI1 expression has been reported to relate to paclitaxel resistance in this disease [49]. Additional pathways were also observed with the NFκB (pNFκB) and JAK/STAT (pSTAT3) pathways being up-regulated. NFκB has been shown to possess a biphasic role in ovarian cancer [50] while paclitaxel treatment of ovarian cancer cells has been reported to activate the STAT3 pathway [51]. The ER-α pathway was again down-regulated as was HER4, however further protein members of the ERBB pathway, EGFR and HER2, were also upregulated late. HER2 expression has been reported to relate to paclitaxel sensitivity in ovarian cancer cells in vitro [52]. These findings are consistent with observations from the gene expression study where several of the proteins used here, mostly within the cell cycle and DDR functional groups, were differentially expressed (Fig. 5).

## Conclusion

The signaling changes induced by carboplatin and carboplatin-paclitaxel indicate pathways activated by the cytotoxic agents and may suggest new targets for therapeutic intervention and potential ways to enhance chemotherapy effects. The changed expression also provides predictive biomarkers of response. If such dynamic changes can be identified in clinical samples during treatment, they may help indicate the responsiveness of the tumor to chemotherapy. Early changes included DDR and cell cycle regulatory proteins associating with tumor volume changes, which were expected. Therefore inhibitors of the cell cycle or the DDR signaling pathway, e.g., CHK inhibitors [53], may merit further testing and assessment. Changes in ESR1 and HER signaling were also observed, as were late changes in EMT and invasion markers, and these require further investigation to assess their generality.

### Availability of supporting data

All protein expression values analysed here are available as Additional file 1 (Protein expression matrix). Additionally, calculated log fold change values of differentially expressed proteins for each condition are available as Additional file 3 (Differential protein expression).

Gene expression values shown in Fig. 5 are taken from [29] and are available from the Gene Expression Omnibus (GEO) with accession number GSE49577 (http://www.ncbi.nlm.nih.gov/geo/query/acc.cgi?acc=GSE49577).

## Additional files

Additional file 1: Protein expression matrix. Protein expression matrix of raw data of 49 proteins/phosphoproteins assayed in 166 xenograft samples of the dataset. (XLSX 224 kb) Additional file 2: Assessment of biological replicate concordance. Number of replicates for all tested conditions (xenograft/treatment/day) is shown. Correlation coefficients were calculated for all possible pairs of biological replicates and averaged (mean replicate r) using expression values from all detected proteins within each condition. (XLSX 42 kb) Additional file 3: Differential protein expression. Log fold changes of differentially expressed proteins over 14 days are shown in 4 worksheets, each for a xenograft-treatment condition: carboplatin treated OV1002; carboplatin treated HOX424; carboplatin-paclitaxel treated OV1002; carboplatin-paclitaxel treated HOX424. (XLS 57 kb)

## Abbreviations

AQUA: automated quantitative analysis
DDR: DNA damage response
EMT: epithelial to mesenchymal
RTV: relative tumor volume
